# Pathogenesis of Herpes Stromal Keratitis: Immune Inflammatory Response Mediated by Inflammatory Regulators

**DOI:** 10.3389/fimmu.2020.00766

**Published:** 2020-05-13

**Authors:** Li Wang, Runbiao Wang, Chuyang Xu, Hongyan Zhou

**Affiliations:** ^1^Department of Ophthalmology, China–Japan Union Hospital of Jilin University, Changchun, China; ^2^Department of Ophthalmology, Jilin City Central Hospital, Jilin, China

**Keywords:** stromal keratitis, immune response, herpes simplex virus (HSV-1), inflammation, pathogenesis

## Abstract

Herpes stromal keratitis (HSK) is one of the primary diseases that cause vision loss or even blindness after herpes simplex virus (HSV)-1 infection. HSK-associated vision impairment is predominantly due to corneal scarring and neovascularization caused by inflammation. In the infected cornea, HSV can activate innate and adaptive immune responses of host cells, which triggers a cascade of reactions that leads to the release of inflammatory cytokines, chemokines, microRNA, and other regulatory factors that have stimulating or inhibitory effects on tissue. Physiologically, host cells show homeostasis. In this review, we summarize the factors involved in HSK pathogenesis from the perspective of immunity, molecules, and pathological angiogenesis. We also describe in detail the pathogenesis of chronic inflammatory lesions of the corneal stroma in response to HSV-1 infection.

## Introduction

**Figure 1:**
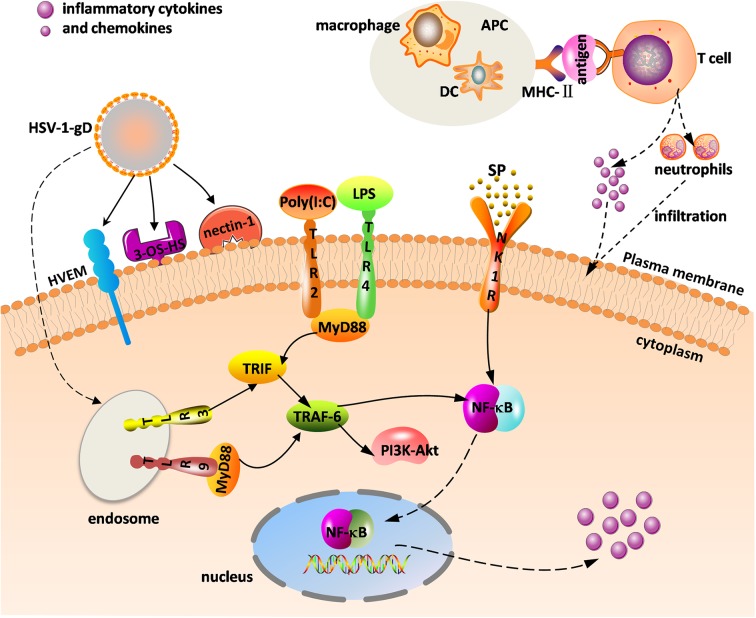
Innate and adaptive immune response in herpes stromal keratitis (HSK) in response to herpes simplex virus (HSV-1) infection. The combination of gD and its receptors [e.g., herpesvirus entry mediator (HVEM), 3-OS-HS, and nectin-1] facilitates virus fusion. HSV-1 and virus analogs [e.g., ploy(I:C), LPS] bind to Toll-like receptors (TLRs) to promote intracellular signaling. Major histocompatibility complex II (MHC-II) transmits the antigenic information on the surface of antigen-presenting cells (APCs) [e.g., dendritic cells (DC), macrophage] to T cells, and activated T cells promote the release of inflammatory factors and neutrophils. The combination of substance P (SP) and the neurokinin-1 receptor (NK1R) activates nuclear factor κB (NF-κB) to increase inflammatory cytokine and chemokine levels.

Herpes simplex virus (HSV) mainly consists of HSV-1 and HSV-2, and the former is the primary pathogen of herpes stromal keratitis (HSK). HSV-1-induced corneal infection can cause a series of subsequent immune responses. HSK is currently considered a potentially blinding disease. The main clinical manifestations include corneal opacity, edema, corneal scarring, and neovascularization (NV), which can lead to irreversible vision impairment and blindness. HSV-1 in an infected cornea initiates virus replication in the corneal epithelial cells and binding of virus to Toll-like receptors (TLRs) on the cell surface that induces a cascade of innate immune responses and signal pathway responses, stimulating the production of inflammatory cells, cytokines, and chemokines that gradually infiltrate into the stroma. These inflammatory cells including neutrophils, dendritic cells (DCs), natural killer (NK) cells, and macrophages, reportedly help clear HSV-1 from the cornea during the initial infection (1). Importantly, NK cells limit viral replication via the production of type 1 interferons (IFNs) from corneal epithelial cells, resulting in enhanced antiviral activity (1). As antigen-presenting cells (APCs), DCs and macrophages can phagocytize viral particles and infected cells. Once the major histocompatibility complex II (MHC-II) transmits information presented by APCs to the surface of the helper T cells (e.g., Th1 and Th17 T cells), helper T cells are stimulated to produce cytokines and chemokines, eventually triggering adaptive immune responses that is a kind of delayed-type hypersensitivity (DTH) (Figure 1) (2). Th1 and Th17 T cells are two categories of CD4^+^ T cells, which are important in mediating the inflammatory immune response of HSK.

HSV-1 is a neurotrophic virus that is latent in the trigeminal ganglion (TG) following corneal primary infection. Reactivation of latent virus occurs in the context of hypoimmunity and autoimmune disorders. The virus travels back to the corneal epithelium along the axon, causing recurrent HSK. This chronic immune disease mediated by T lymphocytes is caused by viruses and corresponding antigens. In addition to cytokines and chemokines involved in HSK development, neuropeptide substance P, glycoproteins, microRNAs, and other mediators participate in the pathological immune response of HSK. All of these regulators play dual roles of inhibiting and promoting disease during HSK pathogenesis. The purpose of this review is to provide a comprehensive overview of the pathogenesis and progression of HSK from different viewpoints including cellular immunity, inflammatory factors, and mediating molecules to better clarify the immunopathological process of HSK and provide an effective reference for its treatment.

### HSV-1 Entry Into Cells

HSV-1 is a member of the alpha subfamily of herpesviruses and is a linear, double-stranded DNA virus surrounded by an icosahedral capsid. The capsid is composed of two layers of teguments: the inner one is composed of mRNAs and membrane proteins, and the outer tegument is covered by a lipid bilayer envelope containing various glycoproteins and membrane proteins (3). Glycoproteins encoded by the virus mainly include gB, gC, gD, gE, gG, gH, gI, gJ, gK, gL, gM, and gN (4–6). For most herpesviruses, only four or five of these glycoproteins may be involved in viral cell entry. These representative glycoproteins mainly include gB, gC, gD, gH, and gL, and their important role is to bind the virus to heparin sulfate (HS) on the cell surface (5). HS also simultaneously engages host receptors to gain HSV-1 entry into the cell.

HS is a glycosaminoglycan widely present on the cell surface and extracellular matrix (ECM) (7), which can interact with receptors and act as an attachment and receptor for HSV-1 to enter infected host cells (5, 8, 9). HSV-1 binding to HS is the primary way to trigger virus entry. Depending on the different types of viral glycoprotein, there is a corresponding glycoprotein receptor on the cell surface. Taking gD as an example, the virus can fuse with host cells by combining gD with gD receptors, such as 3-OS-HS, herpesvirus entry mediator (HVEM), or nectin-1, which facilitates capsid penetration into the cytoplasm (Figure 1) (9). The capsid is then transported along microtubules to the nucleus, where viral DNA is released for virus gene replication and eventual production of viral progenies that can infect other nearby cells (9). Endocytosis is another pathway by which the virus accesses cells. An earlier study demonstrated that increased HS expression in the early stage of HSV-1 infection can promote virus attachment to cells. However, there is an unexpected decrease in HS later in the infection (10). We believe that decreased HS expression in this stage is mainly related to the HS-degrading enzyme heparanase (HPSE), which can clear HS from the surface of infected cells and further facilitate virus release (8). HPSE activation promotes viral egress from the corneal epithelium and contributes to HSK pathogenesis. Moreover, the virus can retrogradely transport from the corneal epithelium to the TG. This establishes latency, and reactivation of HSV-1 in the TG in some certain conditions results in recurrent HSK. Taken together, HSV entry, fusion, and egress are important conditions for HSK initiation.

### HSK Clinical Characteristics

Stroma infection by HSV-1 is termed HSK, and the most serious clinical manifestation is a corneal ulcer. HSK is characterized by chronic inflammation of the corneal stroma, including cell infiltration, and NV. Recurrent HSK can result in long-term visual impairment due to the immune inflammatory response and is one of the leading causes of blindness.

Patients with HSK usually present with symptoms of redness, photophobia, tearing, eye pain, and blurred vision (11). Based on different clinical manifestations, HSK is divided into two clinical types: non-necrotizing stromal keratitis and necrotizing stromal keratitis. Non-necrotizing stromal keratitis, also known as immune stromal keratitis, is most common in patients with disciform keratitis. We can observe disc edema of the central corneal stromal without inflammatory cell infiltration and NV, as well as wrinkling of the Descemet membrane on slit lamp examination. Necrotizing stromal keratitis manifests as single or multiple yellow-white necrotic infiltrations in the corneal stroma. NV of the corneal stroma generally extends from the peripheral cornea to the infiltrating site of the central stroma. As the lesion progresses, necrotizing stromal keratitis can cause corneal thinning, ulceration, and even perforation. Overall, HSK is the result of an immune-mediated inflammatory response that leads to the development of corneal thinning, focal stromal opacity, corneal NV (CNV), corneal scarring, and potentially blindness (11).

## The Role of T Cells in HSK

Studies in animal models of HSK have reported that T cells [e.g., CD4^+^ T cells, CD8^+^ T cells, and regulatory T cells (Treg)] are the principal orchestrators of immune pathological lesions, and these T cells play different roles and jointly promote HSK pathogenesis (12, 13). CD4^+^T cells have a variety of phenotypes including helper and regulatory T cells (represented by Th1, Th2, Th17, etc.). CD4^+^ Th1 cells mainly secrete pro-inflammatory cytokines IFN-γ and interleukin (IL)-2, which are primarily expressed in the early stage of HSK (14). Th2 cells produce protective cytokines IL-10 and IL-4 that are involved in the process of corneal repair. CD4^+^ T cells appear on day 7 after HSV-1 infection and peak on day 10 post-infection (dpi) (15). HSK usually occurs 7 dpi and peaks at 14–21 dpi (11). This indicates that the appearance of CD4^+^ T cells is accompanied by clinical symptoms of HSK and adaptive immune responses. Within 24 h of infection, viral replication in the cornea triggers the innate immune response. Meanwhile, there is an influx of inflammatory cells including NK cells, DC, macrophages, and neutrophils, with neutrophils being the most important (11). Neutrophils account for 70–80% of leukocytes in the process of chronic inflammatory infiltration (16). There are two neutrophil chemotactic gradients in the immunopathological course of HSK. One appears when the virus replicates in the corneal epithelium. The other appears in the late stage of infection and is closely related to CD4^+^ and CD8^+^ T cells that are essential for the establishment and maintenance of the second chemotactic gradient (17). Sherrie et al. demonstrated that increased levels of inflammatory cells such as neutrophils and DC generally infiltrate the cornea in the presence of activated CD4^+^ cells (18). It has been suggested that autophagic DCs are involved in the activation of CD4^+^ T cells and play a catalytic role in the HSK immune response. In DC autophagy-deficient (atg5^fl/fl^ CD11c-cre) mice, both inflammatory cell infiltration and cytokine expression were decreased after the destruction of activated CD4^+^ T cells, indicating that HSK severity decreased (19). The B and T lymphocyte attenuator (BTLA), a co-receptor expressed in T cells, can weaken the Th1 cell response by downregulating the number of CD4^+^ T cells, which impairs DTH, thereby reducing the incidence of HSK lesions (15). CD4^+^ cells are therefore important in promoting inflammatory cell secretion into the cornea and induce the immune inflammatory response. In a study of 12 HSK patients, virus-specific T-cell infiltration was found in the cornea, suggesting that the immunopathogenic T-cell response is directed to the initial virus but is not related to human corneal autoantigens, further demonstrating the importance of the T-cell immune response in HSK (20).

Researchers use C57BL/6 (HSV sensitive) and BALB/c (HSV resistant) mice with CD4 or CD8 gene knockout (KO) to test the roles of different T lymphocyte cell subsets in recurrent HSK. Their findings show that both CD4^+^ and CD8^+^ T cells contribute to stromal inflammation in HSK, depending on the host gene background (21). Inflammatory cell infiltration in the cornea of CD4^+^ T cell-deficient mice peak at 14 days after HSV-1 infection, and then gradually decrease until 35 dpi, indicating that CD8^+^ T cells may be involved in the inflammatory response and promote the infiltration of inflammatory cells dominated by neutrophils to the cornea in the absence of CD4^+^ T cells (22). In addition to the pro-inflammatory effect of CD8^+^ T cells, there is evidence that CD8^+^ tissue-resident memory T cells (T_RM_ cells), a subset of memory CD8^+^ T cells, have a protective effect and inhibit pathogen infection (23). Moreover, CD8^+^ T cells can be latent in the TG and suppress the virus reactivation there (24). To confirm this observation, the same research team used different test methods and came to the conclusion that this antigen-specific CD8^+^ T-cell response in the TG did not enhance and maintain the latency; rather, CD8α DCs play a key role in establishment and maintenance of HSV-1 latency, thus reducing disease severity (24, 25). HSV-1 reactivation in the TG is a predominant cause of corneal blinding scarring (26). Similarly, HSV-1 infected the cornea of CD4^+^ and CD8^+^ T-cell-deficient mice, and CD8^+^ T-cell-deficient mice showed more severe lesions than CD4^+^ T-cell-deficient mice, suggesting that CD8^+^ T cells have a protective effect against the immunopathological response of HSK (27). Compared with CD4^+^ T cells, the CD8^+^ T-cell response is transient and plays a minor role in terms of the pro-inflammatory effect (28). This may be because CD8^+^ T cells are prominently latent in the infected TG and rarely present in the corneal stroma.

In addition, CD4^+^ Th17 cells can secrete the cytokine IL-17 that induces neutrophil infiltration into the cornea and are mainly involved in the late stage of HSK, whereas Th1 cells predominate during the early stage (29). Tregs, another important subtype of CD4^+^ T cells in HSK pathogenesis, express transcription factor Foxp3, and DNA demethylation of Treg-specific demethylation region (TSDR) is the key to stable Foxp3 expression. Pro-inflammatory cytokines such as IL-2 and IL-6 can influence Treg stability by regulating the epigenetic status of the Foxp3 gene or its post-translational modification, which can lead to the production of ex-Tregs during HSK development. ex-Treg is a kind of Treg that once expressed Foxp3 but now lacks its expression. Under normal physiological conditions, there are no ex-Tregs in the cornea. In the inflammatory state, unstable Tregs can be transformed into ex-Tregs with the same pathogenicity as the effector CD4^+^ Th1 cells, thus aggravating the severity of HSK inflammatory lesions (12). The two most common types of Tregs are natural Tregs (nTregs) and induced Tregs (iTregs). nTreg comes from thymus and participates in preventing autoimmunity. Stabilized iTregs can effectively prevent the development of HSK lesions; they are generated from naive cells and induced *in vitro* (30). Tregs express IL-6R, and inhibition of the IL-6R signal pathway may contribute to Treg stability. IL-6 can induce the expression of DNMT1 (DNA methyltransferase 1) that methylates Foxp3, which directly downregulates Foxp3 gene expression. Azacytidine and retinoicacid enhance the suppressive function and stability of Tregs by inhibiting DNMT activity, which reduces HSV-1-induced corneal damage (31, 32). IL-6 plays an important role in regulating the balance between Tregs and Th17 cells by promoting Th17 differentiation and inhibiting Treg differentiation (33). Moreover, CD4^+^CD25^+^ Tregs can also protect the cornea from more severe lesions, and depletion of CD4^+^CD25^+^ Tregs can accelerate HSK progression (34). A recent study reported that treating the virus-induced inflammatory response with anti-IL-27 antibody can increase the numbers of CD4^+^Foxp3^+^Tregs, ameliorating tissue damage in the cornea (35). Collectively, Tregs play a protective role in maintaining homeostasis, enhancing immunological tolerance, and preventing autoimmune diseases. Tregs are regarded as protective immune regulatory mediators that can control the release of inflammatory cytokines and chemokines, as well as defend against viral invasion of the cornea (36).

## The Role of Cytokines in HSK

When HSV-1 infects the corneal epithelium, it spreads to the stroma or viral particles latent in the corneal stroma, and TG is activated, triggering the innate immune response followed by the adaptive immune response. This process induces the production of pro-inflammatory and anti-inflammatory cytokines. HSK occurs when the balance maintained by pro-inflammatory and anti-inflammatory mechanisms *in vivo* is shifted to an inflammatory state. The role of critical cytokines will be elaborated on from the perspectives of pro-inflammation and anti-inflammation.

### Regulation of HSK by Pro-inflammatory Cytokines

IL-17 is responsible for the immune-inflammatory response of HSK; this crucial pro-inflammatory cytokine can stimulate the production of pro-inflammatory cytokines and neutrophil chemotactic factors by regulating the secretion of corneal stromal fibroblasts (37). To date, IL-17 family members have been identified, including IL-17A to IL-17F, of which IL-17A is the most familiar one and can be detected in corneal epithelium (38). Treatment of an HSK mice model with an anti-IL-17 antibody can effectively suppress the DTH response and significantly reduce lesion severity (39). Xia et al. speculated that the pro-inflammatory mechanism of IL-17 may be promoting the DTH response and upregulating tumor necrosis factor (TNF)-α expression (39). IFN-γ is one of the factors regulating IL-17 expression in the cornea. IFN-γ can activate the innate immune system, leading to increased secretion of various cytokines, and chemokines. Molesworth-Kenyon and colleagues reported that high expression of IL-17 occurs in the cornea of IFN-γ KO mice, indicating that IFN-γ negatively regulates IL-17 expression (40). They performed reverse transcription polymerase chain reaction (RT-PCR) and found that IL-17 mRNA is increased within 24 h after HSV-1 infection and subsequently remains at a lower level during 7 dpi (40). However, Suryawanshi et al. observed that there were two waves of IL-17: the first peak was 2 dpi and the second gradually increased during 7–21 dpi (29). It can be concluded that IL-17 is involved in the whole immune response of HSK, again emphasizing the importance of IL-17. Previous RT-PCR studies showed IL-17R in corneal fibroblasts in the human cornea. Binding of IL-17 to TNF-α or IFN-γ can promote the production of IL-6, IL-8, and macrophage inflammatory protein 3 (MIP3)-α, which accelerates the development of inflammation in HSK (37).

TNF-α is a pro-inflammatory cytokine produced mainly by Thl cells as well as by macrophages (41). IL-1 and TNF-α jointly promote the occurrence of the inflammatory lesions in recurrent HSK, and TNF-α can indirectly advance the properties of IL-1 (42). Contrary to our previous view, Minagawa et al. reported that TNF-α plays an antiviral role in primary and recurrent acute HSV-1 infection (43). IL-1 is expressed in the HSV-1-infected corneal epithelium, which can synergistically promote the infiltration of polymorphonuclear leukocytes into the cornea together with IL-6, triggering a series of inflammatory cascade reactions and inducing HSK (44). IL-6 is a critical pro-inflammatory cytokine that can significantly increase the chemokines MIP-1α and MIP-2, thereby recruiting neutrophils to infiltrate the infected cornea (45). After corneal infection with HSV-1, IL-17, together with TNF-α, can increase the secretion of IL-6, which is involved in the immunopathology, and NV of HSK (37). Consistent with the above experimental results, Hou et al. found that IL-17F could upregulate IL-6 levels and plays a major role in the earlier acute stage of HSK (46). Others observed that drug treatment of HSV-1-induced HSK can significantly reduce IL-6 levels in the mouse cornea (47). Nevertheless, under certain circumstances, IL-6 is considered to be an anti-inflammatory cytokine (48). More interestingly, IL-6 is not an essential cytokine for recurrent HSK (49). In addition to its role as a pro-inflammatory cytokine, IL-6 is also involved in CNV formation, which will be detailed in the following sections.

### Regulation of Anti-inflammatory Cytokines in HSK

In addition to the minor effect mentioned above, IFN-γ produced by NK and Th1 cells plays a protective role in HSK pathogenesis (50). IFN-γ is also known as a Th1-derived cytokine and regulates the neutrophil invasion into the cornea. IFN-γ is generally detected on 3 dpi in the inflamed sites of corneal stroma and lasts until 12 dpi (51). The treatment of HSK with antisense oligonucleotides targeting IFN-γ mRNA (IFN-γ-ASON) *in vitro* and *in vivo* obviously improves the incidence of HSK (52). However, another study demonstrated that IFN-γ has a dual role in the HSV-1-induced immune response. IFN-γ plays a protective role in the early stage of HSK due to inhibition of the production of Th17 cells and limiting viral replication in the cornea, but IFN-γ accelerates the immunopathological process in the late stage in coordination with Th17 cells (29). Additionally, both IFN-γ and TNF-α are protective after HSV-1 infection and inhibit reactivation in latency, as shown using IFN-γ and a TNF-α KO mouse model (53). More importantly, IFN-γ can maintain the latency of the virus in the TG, effectively inactivating it (54). IFN-γ can prevent viral replication in primary and recurrent HSK and reduce the incidence of corneal edema. Compared with mice with primary HSK, the IFN-γ KO mice showed more severe clinical manifestations in recurrent HSK (55). This shows that IFN-γ exerts both antiviral and pro-inflammatory effects in different stages of recurrent HSK (56). IFN-α/β is classified as type I IFN that can trigger a variety of antiviral pathways *in vivo* (e.g., RNase L, PKR) and also maintain the maturation and activation of DCs and T lymphocytes (57). IFN-α/β can control HSV-1 replication and reactivation of the virus in the TG during acute ocular infection, thus controlling the transmission of the virus from the TG to the cornea (57). IFN-γ and IFN-β can synergistically dampen virus replication *in vivo* and *in vitro*, which results in enhancing antiviral effects and resisting viral infection (58).

Studies in mouse models of HSK have revealed that Tregs and IL-10 independently play protective roles in the HSV-1-induced inflammatory response. Depletion of IL-10 and/or Tregs can potentiate the severity of the HSV-1 infection in the cornea. One anti-inflammatory mechanism of IL-10 is to suppress the proliferation of CD4^+^ and CD8^+^ T cells and the production of inflammatory cytokines and chemokines such as IL-2, IL-6, and MIP-1α (59, 60). The other mechanism is that IL-10 can inhibit the DTH response (61). Based on the reduction of infiltration of neutrophils into the cornea by IL-10 treatment, we can conclude that IL-10 can significantly reduce the incidence of HSK (60, 61). IL-10 is derived from corneal epithelium cells and fibroblasts and is an endogenous immunosuppressive cytokine (62, 63). Some researchers have proposed treating HSK by upregulating IL-10 (64). To date, we are familiar with two major anti-inflammatory cytokines: IL-10 and IL-4. IL-4 is also known to downregulate inflammatory cytokine expression and protect against HSV-1-induced corneal scarring. More importantly, IL-4 can reduce viral replication in the cornea (65, 66). IL-4 can also promote immune responses in the context of allergy or parasitic infections. Overall, pro-inflammatory and anti-inflammatory cytokines maintain a balance during HSK pathogenesis. Both pro-inflammatory and anti-inflammatory effects depend on the particular condition, and some cytokines may have dual roles. There are more inflammatory cytokines that need to be identified to better understand their role in HSK pathogenesis.

## The Role of Chemokines in HSK

Chemokines have chemotactic specificity and can recruit neutrophils to gather at the site of lesions, leading to the development of diseases. These chemokines are thought to have pro-inflammatory properties. In contrast, some chemokines are homeostatic and can control disease progress (67). Chemokines bind to G protein-coupled receptors, which induce conformational changes, thereby activating intracellular signal transduction pathways and triggering the inflammatory response. Most chemokines have conserved cysteines in amino acid sequences. According to the difference of the first two cysteine locations, chemokines can be divided into four families: CXC, CC, C, and CX3C. The two major chemokine types are CXC and CC (68). A variety of chemokines are involved in the pathology process of HSK, including CCL2, CCL3, CXCL1, and CXCL10.

CXCL1, also known as keratinocyte-derived chemokine (KC), is the only chemokine produced by the central corneal epithelium and is considered a neutrophil chemoattractant (69). CXCL1 contributes to neutrophil migration to the cornea early after infection, especially on 3 dpi. Bryant-Hudson et al. further demonstrated that CXCL1 and CXCL2 are not involved in clearing the virus (70). The recurrence rate in the anti-CXCL1 treatment group was significantly lower than the other two groups in a mouse model of recurrent HSK, indicating that CXCL1 is essential to recurrent HSK (49).

CXCL10 has a protective function and is homeostatic, and this unique chemokine is expressed in the TG and epithelial cells. The receptor of CXCL10 is CXCR3, and it participates in the Th1 response (67). Based on the research of Srivastava and colleagues, we speculate that CXCL10 can control the inflammatory response via two ways: (1) involvement in the immunity of antiviral CD8^+^ T cells in the infected cornea and TG and (2) elevating the level of protective HSV-specific CD8^+^ T cells in infected tissues. As a result, mice lacking CXCL10/CXCR3 have shown more severe lesions (71). Previous studies also demonstrated that the absence of CXCL10 increases the influx of inflammatory cells to infected corneas and the expression of angiogenic factors, which exacerbates HSK severity (72). Additionally, Srivastava et al. reported that boosting the number and function of HSV-specific CD8^+^ T cells by CXCL10 in the cornea and TG can protect the cornea against HSV-1 infection (73). In line with these results, enhancing CXCL10 levels can bolster the number of effector memory CD8^+^ T cells (T_EM_) and tissue-resident memory CD8^+^ T cells (T_RM_) in the TG, to inhibit viral reactivation and reduce the incidence of recurrent HSK (74). However, it is interesting that lack of CXCL10 weakens the severity of HSK and that CXCL9 was detected in the absence of CXCL10, indicating that CXCL10 has dual pro-inflammatory and anti-inflammatory roles, and CXCL9 accelerates HSK progression (75).

CXCL9 comes from resident corneal cells and neutrophils and can aggregate neutrophils and trigger an inflammatory response (76). The presence of TLR9 and type IIFN signaling is a precondition for CXCL9 and CXCL10 expression in the cornea following HSV-1 infection (77). The pro-inflammatory role of CXCL9 is associated with T-cell trafficking into the cornea, as decreased infiltration of CD4^+^ T cells is found in the mice lacking CXCL9 (78). Conversely, chemokine levels should be reduced *in vivo* in the absence of CD4^+^ T cells post HSV-1 infection, while the number of CXCL9 and CCL5 are not reduced significantly after 3 dpi in the context of CD4^+^ T-cell depletion (79). CXCL9 and CXCL10 bind to the same receptor CXCR3. Mice deficient in CXCR3 and CXCR5 manifest less severe diseases, indicating that both receptors play crucial functions in the pathological immune response to HSK (80).

CCL2, also known as monocyte chemoattractant protein (MCP-1), is a critical mediator of inflammatory monocyte recruitment into the cornea (81). The corneal stromal cell is the primary source of MCP-1 and CCL5, which in turn elicits CD4^+^ T-cell migration toward corneal lesions, exacerbating stromal inflammation (82). Moreover, CCL2 production is driven by IFN-α, which recruits inflammatory cells to infected sites to induce inflammatory responses (81). Nevertheless, the results of a previous study demonstrated that MCP-1 is not the major inflammatory chemokine in the HSV-1-infected cornea. Decreased macrophage infiltration was observed in MCP-1 KO mice, but overexpression of pro-inflammatory cytokines and other chemokines (e.g., MIP-2/CXCL2 and MIP-1α) was associated with more severe lesions and angiogenesis, suggesting that MIP-2 plays an important role in the absence of MCP-1 (83).

MIP-1α/CCL3 is another regulator of C-C chemokines that functions as a protective chemokine by protecting against viral infection in the cornea (84). MIP-2 and MIP-1α are generated following the stimulation by IL-17, which is further proof for the pro-inflammatory role of IL-17 in HSK pathogenesis (40). However, an earlier study had reported that MIP-2 and MIP-1α were pivotal promoters of neutrophil influx into the corneal infection site (40). From the perspective of the response of MIP-1α to inflammatory cells, it can be concluded that MIP-1α also has a dual function of promoting and inhibiting the development of HSK. CCL5 was also detected in the HSV-1-infected cornea where it binds to the receptor CCR5 to mobilize neutrophil migration to the cornea (85). In conclusion, the expression of chemokines in the HSV-1-infected cornea largely influences inflammatory cell infiltration into the cornea.

## The Role of Glycoproteins in HSK

Glycoprotein K (gK) is one of the 12 known glycoproteins encoded by the virus, and HSV-1 is covered by glycoproteins. gK is mainly involved in viral entry into corneal epithelial cells, membrane fusion, the intracellular spread of virions, and virion egress (86). Many studies support the viewpoint that gK plays important roles in viral replication and corneal scarring (6, 87). Lack of gK can reduce virus transmission to the corneal epithelium and TG, which prevents the establishment of latency and thus inhibits viral replication and reduces the development of corneal nerve damage (88, 89). The cause of corneal neuroinvasiveness appears to be the amino terminus of gK (90). Researchers investigated the effect of HSV-1 infection in the mouse cornea by overexpressing gK. They found that overexpression of gK increased the severity of corneal scarring by altering how the virus bound to the receptor by modulating the expression of HSV-1 receptors, such as 3-OS-HS, HVEM, and nectin-1 or nectin-2 (91). One hypothesis is that binding of gK to signal peptide peptidase (SPP) exacerbates corneal scarring and leads to blindness (87, 92). The pathogenicity of this combination is mainly manifested as increased viral replication and the establishment of latency, as well as enhanced numbers of viral transcripts in the cornea and TG (87). Furthermore, we have realized that gK is the only glycoprotein that binds SPP *in vitro* (92), and recent research further demonstrated that the combination of gK and SPP can occur *in vivo* (87). Therefore, the importance of gK in the pathogenesis of HSK is compelling.

It was also found that CD8^+^ T cells, usually considered as CD8^+^CD25^+^ T cells, play a decisive role in accelerating corneal scarring in gK-immunized mice (6, 93). A particular peptide is gK T-cell-stimulatory region as well as CD8^+^ T-cell epitope, which is named gK 8mer. We conclude that gK 8mer participates in accelerating corneal scarring by inducing the CD8^+^ T-cell response (94, 95). Reducing of the level of CD8^+^ T-cell infiltration in the cornea of gK-immunized mice can attenuate corneal scarring (96). Additionally, the property of antibody-dependent enhancement (ADE) of gK contributes to potentiating HSV-1 infection in corneal stroma compared with glycoprotein D (gD) (97), so gD plays a protective role in HSK.

Researchers extracted corneal HSV-1 isolates from 178 HSK patients for genetic testing of gG, gI, and gE. There was a high glycoprotein detection rate in HSV-1 isolates, but the clinical manifestation of HSK was independent of gG or gI genotype (98). Similarly, a case report on HSV-1-induced keratitis described a significant finding that gG and gI were detected in tear samples from patients with bilateral necrotizing herpes simplex keratitis (99). Unfortunately, the correlation between HSV-1 genotype and clinical manifestations is not very clear. In a word, antibody titers such as gB, gD, gK, and gE can be detected in the sera of patients with HSK, and gD and gK are the most widely studied. The mechanisms by which gK exacerbates HSK can be summarized as follows: (1) the combination of gK and SPP, (2) the CD8^+^ T-cell response induced by gK, (3) the involvement of gK in axonal transport, and (4) the expression of receptors regulated by gK. Blocking any of the above gK-induced processes can reduce HSK severity and inhibit viral replication. Thus, gK is an important determinant in viral infectivity and corneal manifestation in HSK.

## The Role of Substance P in HSK

Substance P (SP) is a neuropeptide substance produced by nerve fibers and APCs in the corneal stroma during the clinical phase. SP is a pro-inflammatory mediator involved in stimulating the production of inflammatory cytokines and chemokines (100). Intraperitoneal injection of SP increases inflammatory cell levels in mice, leading to more severe diseases (101). The level of SP in the HSV-1-infected corneal stroma is in direct proportion to HSK severity. With higher amounts of SP, more inflammatory cells and CD4^+^ T cells are produced (100). The reason for this pro-inflammatory effect is that the binding of SP to the neurokinin-1 receptor (NK1R) activates the nuclear factor-κB (NF-κB) signal pathway, triggering neutrophil influx into the inflamed cornea (102). NK1R is an important receptor of SP and is expressed in corneal epithelium. The affinity of SP and NK1R determines the rate of HSK progression. In the early stage of infection, the corneal opacity and angiogenesis of HSK are aggravated in mice lacking in NK1R, which is contrary to the previous view that absence of NK1R can reduce the severity of eye diseases (103). The number of sloughing of apical corneal epithelial cells and viral load increase after HSV-1 infection, and CD4^+^ T cells are upregulated, which promotes the infiltration of inflammatory cytokines and chemokines, such as MIP-2, MIP-1α, and MCP-1 (103). Studies have revealed that increased release of chemokine and chemokine receptors is due to binding of SP to NK1R and subsequent activation of the NF-κB pathway (104). Moreover, with the exception of pro-inflammatory properties, SP is also involved in wound healing. Activation of the PI3K/Rac1/RhoA pathway following SP binding with NK1R can promote keratocyte migration, which promotes stromal wound repair. SP also stimulates keratocytes to produce IL-8, which further contributes to keratocyte migration and leads to an acute inflammatory response (105). SP also plays a role in promoting corneal angiogenesis in humans and mice (106). NK1R antagonists can significantly reduce the extent of CNV in both alkali burn and suture-induced NV models and downregulate SP levels (107). Collectively, SP promotes HSK pathogenesis by expanding the effects of pro-inflammatory responses.

## The Role of microRNAs in HSK

microRNAs (miRNAs) are small, single-stranded, non-coding RNAs that promote mRNA degradation or inhibit protein expression by regulating gene expression (108). miRNAs play crucial roles in viral latency, viral replication, inflammation, and CNV in HSK (108, 109). The involved miRNAs have been reported as miR-155, miR-132, miR-183, miR-23b, miR-326, miR-21, and miR-H2, etc. (108). Of these, miR-155 and miR-132 are the most studied widely in HSV-1-induced corneal infection.

HSV-1 infection of the cornea induces the production of inflammatory cells and SP and also upregulates the level of miR-155, which can lead to overexpression of inflammatory cytokines and chemokines as well as augmented Th1 and Th17 immune responses that contribute to more severe corneal opacity and angiogenesis (110). miR-155 expressed in activated CD4^+^ T cells suppresses the levels of phosphatidylinositol-3,4,5-trisphosphate 5-phosphatase 1 (Ship1) and IFN-γ receptor α-chain (IFN-γRα), and viral replication is not associated with the induction of increased miR-155 (110). Based on this, we propose that miR-155 influences corneal damage via different pathogenic ways and has a prominent role in HSK pathology. Increased expression of miR-132 is detected after HSV-1 infection, and miR-132 promotes CNV (111). It also enhances viral replication by suppression of the p300 transcriptional co-activator, directly regulating the innate immune response (112). In terms of HSK, miR-H2 is mainly expressed in the TG, which can reduce viral latency, and prevent virus reactivation by dampening expression of the ICP0 gene. Conversely, the miR-H2 mutation can increase the activation rate of viral latency and accelerate HSK progression. Therefore, miR-H2 plays a role in attenuating lesion formation during HSK pathogenesis (113). This review lists the typical miRNAs involved in HSK; it is of great significance to target the inflammatory miRNAs to treat this (Table 1).

**Table 1:** Role of microRNAs in herpes stromal keratitis (HSK) (108, 109, 114, 115).

**miRNA**	**Targets**	**Function**	**Effects**
HSV-1-miR-H6	ICP4	Maintains and establishes HSV-1 latency	Anti-inflammation
HSV-1-miR-H2	ICP0	Decreases neurovirulence and reactivation, promotes HSV-1 latency	Anti-inflammation
miR-649	MALT1	Promotes HSV-1 replication	Pro-inflammation
miR-101	GRSF1/ATP5β	Inhibits HSV-1 replication	Anti-inflammation
miR-23a	IRF1	Promotes HSV-1 replication	Pro-inflammation
miR-23b	NF-κB	Inhibits NF-κB expression	Anti-inflammation
	TAB2, TAB3, IKK-α	Inhibits IL-17-associated immune inflammatory expression	
miR-138	ICP0	Promotes host survival and viral latency	Anti-inflammation
miR-155	Ship1/IFNγ-Rα	Promotes CD4^+^ T-cell proliferation, promotes Th1 and Th17 cell immune response	Pro-inflammation
miR-132	Ras-GAP	Promotes VEGF signaling	Pro-angiogenesis
	p300 transcriptional co-activator	Promotes viral replication	Pro-inflammation
miR-21	–	Induces IL-10 production	Anti-inflammation
	Sprouty 2/4	Inhibits VEGF-A and HIF-1α expression	Anti-angiogenesis
miR-146a	–	Inhibits NF-κB and TLR activation, inhibits cytokine production, regulates Tregs to control Th1 response	Anti-inflammation
miR-219	5-Lipoxygenase	Increases SPM^*^ production	Anti-inflammation
miR-208a	–	Induces IL-10 production	Anti-inflammation
miR-146b	–	Negatively regulates IL-8/RANTES	Anti-inflammation
miR-855,miR-491,miR-212	–	Negatively regulates MMP9	Pro-angiogenesis
miR-29	Tbet/Eome	Promotes IFN-γ production by Th1 cells	Pro-inflammation
miR-326, miR-301a	–	Th17 cell production	Pro-inflammation
miR-292-2	Lipoxygenase	Reduces leukotrienes and induces SPM production	Anti-inflammation
miR-10a	Tregs	Stabilizes Tregs	Anti-inflammation
miR-182	Foxo1	Promotes T-cell proliferation	Pro-inflammation
miR-31	FIH-1	Promotes keratocyte glycogen metabolism	Pro-angiogenesis
miR-27a	Sema7A	Enhances VEGF-A levels	Pro-angiogenesis
miR-145	ITGB8	Regulates human corneal epithelial differentiation	Anti-angiogenesis
miR-184	miR-205	Maintains SHIP2 levels in epithelia	Anti-angiogenesis
	VEGF	Inhibits VEGF expression	
miR-204	VEGF, VEGFR2	Reduces VEGF and VEGFR2 expression	Anti-angiogenesis

* *Specialized pro-resolving mediators (SPMs). SPMs are biosynthesized by inflammatory exudates and have anti-inflammatory (limiting further neutrophil infiltration) and pro-resolving (enhancing macrophage clearance of microbial peptides and apoptotic cells) actions*.

## CNV Formation

CNV is a serious clinical manifestation in the advanced stage of HSK and is one of the leading causes of blindness. The cornea, itself, is a transparent, avascular tissue. When the host cornea is infected by HSV-1, neutrophils are stimulated to migrate to the stroma. Under hypoxic conditions, vascular endothelial cells and pericytes are destroyed, and the level of vascular endothelial growth factor (VEGF) is subsequently upregulated and binds with the soluble VEGF receptor (sVEGFR-1). However, upregulation of VEGF-A suppresses sVEGFR-1 expression, which influences binding of VEGF-A and sVEGFR-1 and further promotes the formation of new vasculature (116). VEGF includes four subtypes: VEGF-A, VEGF-B, VEGF-C, and VEGF-D. Among these, VEGF-A is the most prominent in the formation of new blood vessels (117) and is mainly derived from neutrophils in the late stage of HSK (118). The basic steps of CNV formation include (116, 117): (1) leukocyte accumulation at lesions, (2) destruction of the basement membrane of corneal endothelial cells and ECM degradation (3), endothelial cell proliferation, and (4) the formation of new vascular sprouts and lumens. Multiple inflammatory mediators are involved in the process of CNV, including pro-angiogenic factors IL-6, IL-10, IL-17A, CXCL10, miR-132, matrix metalloproteinases (MMPs), and others (119). NV is usually induced when there is an imbalance between pro-angiogenic factors and sVEGFR-1.

HSV-1 infection initiates innate and adaptive immune responses followed by a cascade of inflammatory cytokines, for example, IL-17 production. IL-17A, a member of the IL-17 family, directly induces increased VEGF-A production after viral infection, and indirectly promotes the synthesis of VEGF-A with IL-6 (120). MMP production (MMP-2, MMP-7, and MMP-9) is simultaneously upregulated, which can degrade sVEGFR-1 as well as the ECM, further exacerbating vascular leakage (120). In terms of promoting angiogenesis, IL-17 acts on the cornea as well as on other ocular diseases, such as proliferative diabetic retinopathy and age-related macular degeneration (121, 122). Reducing IL-17 can decrease the severity of CNV. VEGF secretion is induced by stimulating corneal epithelial cells and stromal fibroblast cells with IL-6 post-HSV-1 infection, leading to CNV. As the concentration of IL-6 increases, so does that of VEGF (123). Moreover, CXCR2 is a receptor for pro-inflammatory chemokines and is involved in neutrophil recruitment. The absence of CXCR2 prevents the chemokines from binding to it, which can theoretically reduce neutrophil infiltration and alleviate the severity of corneal damage. However, an interesting study on IL-6 promoting angiogenesis showed exacerbation of HSK in CXCR2 KO mice, mainly manifested as CNV, which is related to increased IL-6 (124). Therefore, IL-6 has both pro-inflammation and pro-angiogenesis roles (125) in HSK pathogenesis. Fibroblast growth factor-2 exerts a stronger and more persistent influence on HSV-1-induced CNV compared with IL-6 (125). As mentioned above, the gene regulator miR-132, as a gene regulator, plays a critical pro-angiogenic role in CNV. Treatment with nanoparticles loaded with miR-132 antagonists can effectively decrease the extent of CNV and diminish corneal edema in HSK (111). Multiple of miRNAs have been implicated in CNV (115), from which we can infer that they have a crucial role in HSK pathogenesis.

Conversely, the cytokine IL-10 suppresses CNV, and treatment of recurrent HSK with IL-10 can attenuate the severity of angiogenesis (61, 63). Likewise, blockade of CXCL10 expression exacerbates angiogenesis in HSK (72). IL-18 inhibits CNV by dampening endothelial cell proliferation, leading to decreased expression of VEGF (126). As such, MMPs mainly including MMP-2 and MMP-9 increase after HSV-1 infection, which plays a catalyst role in NV. In the early stage of viral infection, MMP-9 production is stimulated by inflammatory cells, especially neutrophils, and depletion of neutrophils can reduce MMP-9 activation (127). Both MMP-2 and MMP-9 can degrade the ECM and release pro-angiogenic factors, and the main components of vascular basement membranes are MMP-2 and MMP-9, so both of them are considered as the proteolytic enzymes to degrade the basement membrane, which confirm that MMPs promote CNV (117). MMPs degrade sVEGFR-1, which further contributes to the development of CNV (118). It has also been reported that MMP alone does not promote the formation of new blood vessels (125). Moreover, endothelial receptor Robo4 (R4) can control angiogenesis, and subconjunctival injection of soluble R4 into wild-type mice significantly diminishes the extent of CNV because R4 activates the anti-angiogenic pathway (128). In summary, an imbalance between pro-angiogenic and anti-angiogenic factors results in excessive VEGF, enhancing the development of CNV. Also, CNV occurs when the cornea is exposed to inflammation, hypoxia, and limbal stem cell deficiency (116). If pro-angiogenic factors are effectively controlled during the NV stage, it could reduce the blinding rate of HSK.

## Other Mechanisms

### Regulation of HSK by Signaling Pathways

Polyinosinic–polycytidylic acid [poly(I:C)] is a synthetic analog of viral double-stranded RNA that induces the expression of adherence molecules in corneal fibroblasts by activating multiple signaling pathways including mitogen-activated protein kinase (MAPK), phosphoinositide 3-kinase (PI3K)-Akt, and NF-κB. Of these, NF-κB is the primary signal pathway, and it is downstream of the PI3K pathway (129). NF-κB is a critical transcriptional regulatory factor that regulates the expression of various factors such as cytokines and adherence molecules that influence host immune responses. As a regulator in the signal pathway, NF-κB plays an important role in the immunopathology of HSK. Adherence molecules, including intercellular adhesion molecule-1 and vascular cell adhesion molecule-1, bind to cytokines, and chemokines to induce inflammatory responses. Poly(I:C) also increases TLR3 expression in corneal fibroblasts, which is recognized by the virus and further binds with it, leading to innate immune responses and enhanced secretion of cytokines and chemokines (130, 131). Moreover, poly(I:C) induces the activation of MAPKs, including extracellular signal-regulated kinase, p38, and c-Jun NH2-terminal kinase, all of which mediate the expression of inflammatory cells and adhesion molecules (130). Based on a full understanding of the role of signal pathways in HSK pathogenesis, researchers usually consider each signal pathway as a treatment target, looking for more appropriate drugs to inhibit the inflammatory response. As an example, H-RN is an 11-amino-acid peptide that can suppress the activation of NF-κB and MAPK signal pathway, thereby subsequently diminishing the production of pro-inflammatory cells that are involved in HSK induced by poly(I:C) and lipopolysaccharide (LPS) (132).

### Herpesvirus Entry Mediator

**Figure 2:**
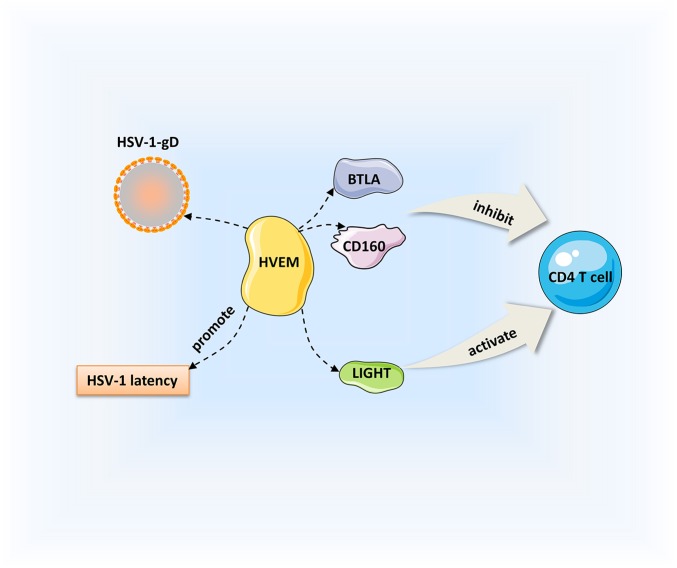
Regulation of HSK by HVEM. HVEM can interact with immunoglobulin ligands [CD160, B, and T lymphocyte attenuator (BTLA)] and the tumor necrosis factor (TNF) ligand [LIGHT]. Binding of HVEM to LIGHT activates CD4^+^ T cells. Conversely, the interactions of CD160 and BTLA with HVEM inhibit CD4^+^ T cell expression. Besides, HVEM can promote the establishment of HSV-1 latency.

Herpesvirus entry mediator (HVEM) is a member of the TNF receptor superfamily that interacts with corresponding ligands, such as immunoglobulin (Ig), TNF, and gD; it is important for viral entry, fusion, and ultimately HSV-1 pathogenesis. The Ig ligands of HVEM include BTLA and CD160, and TNF ligands include LIGHT and lymphotoxin-α (LT-α) (133–135). HVEM is expressed on a variety of cell surfaces, including T cells, B cells, macrophages, and DCs (136). Binding of HVEM to gD contributes to capsid release into the cytoplasm, then the capsids transported to the nucleus where viral replication occurs (9, 137). As a TNF receptor, HVEM can combine with LIGHT to promote the release of TNF receptor-associated factors to the cytoplasm, thus activating the NF-κB signaling and enhancing the production of cytokines and other related factors and initiating immune inflammatory responses (136). Meanwhile, HVEM-mediated NF-κB activation could also improve the survival of neuron reactivation. CD160 is detected on CD4^+^ and CD8^+^ T cells. The interactions of CD160 and BTLA with HVEM inhibit CD4^+^ T-cell activation, whereas it is enhanced by binding of HVEM and LIGHT (135). CD4^+^ T-cell activation triggers the T-cell immune response. Previous studies have demonstrated that HVEM is a pro-inflammatory factor that is a determinate of HSV-1-induced corneal infection pathogenesis (138). Compared with the controls, viral replication and severe symptoms are not observed in HVEM KO mice, and cytokine levels are decreased after HSV-1 infection (138, 139). It is also thought that HVEM in corneal epithelial cells suppresses the production of cytokines and chemokines in response to HSV-1 infection (140). Whether HVEM is pro-inflammatory or anti-inflammatory depends on the interactions with various cell types with different ligands that bind to it. Given that the entry interaction between HVEM and gD does not influence the corneal clinical symptoms, the pathogenic role of HVEM in HSK could be due to its immunomodulatory function in the inflammatory immune process (139). HVEM also contributes to HSV-1 latency in the TG. Latency-associated transcript is a viral gene that is continuously expressed during latency; it promotes HSV-1 reactivation and the establishment of latency by upregulating HVEM expression. HSV-1 latency was significantly decreased in HVEM KO mice, indicating that HVEM plays an important role in this process (141). Moreover, BTLA, LIGHT, and CD160 can interfere with the binding of gD to HVEM, and inhibition of the HVEM/BTLA/LIGHT/CD160 pathway facilitates decreased HSV-1 latency and reactivation (142). The pathogenic mechanism of HVEM is a complicated process including aspects of viral replication, inflammation, the immune response, and viral latency. Based on our current understanding, we can consider HVEM as a treatment target for controlling the key steps of virus entry and immunomodulation (Figure 2).

### The Role of Hypoxia in HSK

HSK is a chronic immune inflammatory disease. Large numbers of neutrophils and CD4^+^ T cells are detected in the HSV-1-infected corneal stroma. In the preclinical stage of HSK, increased expression of hypoxia-related glycolytic genes are detected in the corneas of HSV-1-infected mice, and viral replication in the infected cornea is triggered by innate immune responses during the early stage of infection, which induce neutrophil aggregation (143). Some studies have reported a correlation between hypoxia and inflammation. One of the causes of hypoxia in chronic inflammatory tissues may be the high metabolism of neutrophils, which consumes a large amount of energy, finally leading to tissue hypoxia. Thus, neutrophils are implicated in the development of hypoxia in the immunopathological process of HSK, and the degree of hypoxia is positively correlated with the number of neutrophils infiltrating the cornea. Moreover, tissue hypoxia can induce corneal angiogenesis, which aggravates visual impairment. Hypoxia inducible factors (HIFs) are present in the infected cornea, and blocking HIF-1α and HIF-2α expression with acriflavine can reduce the severity of HSK and induce regression of CNV (143). In summary, hypoxia plays an important role in HSK pathogenesis.

## Conclusion

HSK is one of the leading causes of blindness, and activation of the virus during latency is recognized as the primary cause of recurrent HSK. After infection, the immune response is triggered as HSV enters the corneal epithelium. Homeostasis is maintained by pro-inflammatory and anti-inflammatory mediators *in vivo*. When this balance shifts to pro-inflammatory conditions, the cascade reaction mediated by inflammatory regulatory factors can lead to severe visual impairment, including corneal edema, stromal opacity, and corneal scarring due to inflammatory cell infiltration. In pro-inflammatory conditions, neutrophils can indirectly promote corneal hypoxia and directly aggravate the inflammatory process of corneal lesions. At present, there are few studies on hypoxia for HSK. Hypoxia is related to inflammation and can accelerate the formation of CNV. To date, the understanding of HSK pathogenesis is mainly based on experimental mouse models, and little is known about the effect of HSV-1 virus genotypes on clinical manifestations of human diseases. These studies provide important insights into the roles of host factors in the prevention and immunopathology of HSK. This review elaborates on the various pathogenic factors that may lead to HSK from the immunological and molecular levels. In addition to the cytokines and chemokines required for inflammatory immune responses, this review also emphasizes the important role of glycoproteins, SP, HVEM, hypoxia, and microRNAs in HSK pathogenesis. We believe that more pathogenic factors will be found in future scientific work, such as insulin-like growth factor-binding protein-3, which is present in the epithelium and stroma of HSK developing corneas and protects from aggravation of the lesions (144). It is important to have more in-depth research on hypoxia, microRNA, SP, and other areas to find more effective therapeutic targets for HSK and reduce the blindness rate.

## Author Contributions

All authors listed have made a substantial, direct and intellectual contribution to the work, and approved the manuscript for publication.

## Conflict of Interest

The authors declare that the research was conducted in the absence of any commercial or financial relationships that could be construed as a potential conflict of interest.
